# A High-Gain and Dual-Band Compact Metasurface Antenna for Wi-Fi/WLAN Applications

**DOI:** 10.3390/ma18112538

**Published:** 2025-05-28

**Authors:** Yunhao Zhou, Yilin Zheng

**Affiliations:** School of Electronic and Information Engineering, Soochow University, Suzhou 215006, China; 2228410143@stu.suda.edu.cn

**Keywords:** metasurface antenna, dual-band, artificial magnetic conductor (AMC), high gain, compact, Wi-Fi/WLAN applications

## Abstract

With the rapid development of Wi-Fi 6/6E and dual-band wireless systems, there is an increasing demand for compact antennas with balanced high-gain performance across both 2.4 GHz and 5 GHz bands. However, most existing dual-band metasurface antennas face challenges in uneven gain distribution between lower/higher-frequency bands and structural miniaturization. This paper proposes a high-gain dual-band metasurface antenna based on an artificial magnetic conductor (AMC) array, which has a significant advantage in miniaturization and improving antenna performance. Two types of dual-band AMC structures are applied to design the metasurface antenna. The optimal antenna with dual-slot AMC array operates in the 2.42–2.48 GHz and 5.16–5.53 GHz frequency bands, with a 25% size reduction compared to the reference dual-band U-slot antenna. Meanwhile, high gains of 7.65 dBi and 8 dBi are achieved at 2.4 GHz and 5 GHz frequency bands, respectively. Experimental results verify stable radiation gains across the operation bands, where the total efficiency remains above 90%, agreeing well with the simulation results. This research provides an effective strategy for high-gain and dual-band metasurface antennas, offering a promising solution for integrated modern wireless systems such as Wi-Fi 6, Bluetooth, and MIMO technology.

## 1. Introduction

Recently, wireless local area networks (WLANs) have garnered significant attention. Nearly every residence and workplace in advanced technology markets employs a WLAN, with deployments swiftly expanding in public gathering spaces such as cafés, hotels, and airports. Wireless operators are adopting WLANs for cellular offload as smartphone attachment rates have approached 100 percent [1,2]. China possesses roughly 100 MHz of unlicensed spectrum between 5.15 and 5.725 GHz, while the 2.4 GHz band may support three non-overlapping channels, each 20 MHz wide. The expanded spectrum availability in the 5 GHz and 2.4 GHz bands offers increased network capacity, resulting in fewer competing devices per channel [3]. In comparison, the 5 GHz band has higher speed and larger spectrum [4,5], while the 2.4 GHz band offers stronger diffraction capabilities and wall-penetrating compatibility. For example, sensors in factories and agricultural IoT devices rely on the long-distance coverage of 2.4 GHz, while 40% of IoT devices globally still only support 2.4 GHz [6]. Consequently, the majority of commercial Wi-Fi modules operate in both the 2.4 GHz and 5 GHz frequency bands, hence facilitating a broad spectrum of applications for dual-band antennas.

Given the extensive utilization of dual-band antennas in contemporary communication systems, both academic and industrial sectors have engaged in the comprehensive research and methodical advancement of dual-band antenna technology in recent years. Among diverse dual-band antenna systems, patch antennas have emerged as the predominant selection owing to their distinctive advantages. These antennas exhibit low-profile and lightweight physical qualities, alongside manufacturing benefits such as seamless integration and great cost-effectiveness, rendering them especially appropriate for portable devices and embedded system applications [7]. According to the classification of antenna structure, typical dual-band patch antennas include multiresonant structures [8,9,10], slotted types [11,12,13], parasitic element type [14,15], and many others [16,17,18]. Multiresonant structure patch antennas achieve two distinct resonant frequencies by overlapping smaller patches onto the fundamental patch. Slotted patch antennas attain dual-band functionality by incorporating various current pathways via slots in the patch or ground plane. Parasitic element type antennas incorporate parasitic elements surrounding the primary radiating element, employing electromagnetic (EM) interaction among elements to generate several resonant frequencies. In the design of dual-band antennas, critical characteristics under investigation include bandwidth, antenna dimensions, and radiation gain. The dual-band antennas for Wi-Fi applications should have the advantages of high gain and miniaturization in order to guarantee a high signal strength along with a modular integrated design. Traditional dual-band patch antennas struggle to achieve an optimal equilibrium between gain and dimensions [11,12,13,19,20,21,22], necessitating the introduction of novel structural designs to mitigate this issue.

Metasurfaces are periodic structures composed of quasi-two-dimensional artificial elements in subwavelength scale that can flexibly manipulate EM characteristics, including amplitude, phase, frequency, and polarization [23,24,25]. Benefiting from their flexible EM control capabilities, metasurfaces have been widely applied in antenna design in recent years to achieve low-profile, wideband, high-gain, and multifunctional characteristics, providing innovative design solutions for modern wireless communication systems [26]. Considering the roles of the metastructure, there are two typical implementation approaches for metasurface antennas: one is the metasurface-assisted antenna, and the other is the metantenna [27]. For metasurface-assisted antennas, metasurface structures are applied to replace the ground plane or are placed above and around the radiation structures, thereby enhancing antenna performances such as radiation gain, bandwidth, axial-ratio bandwidth, aperture efficiency, and so on. In this case, the metasurface and antenna are designed separately and then combined. Metasurface serves as an auxiliary performance-enhancing component for the antenna, which means that the antenna can still operate if the metasurface is removed, just with attenuated performances. With regard to metantennas, metasurface structures are directly used as the radiator aperture of antennas, rather than only as auxiliary substrates or superstrates, inspiring the ultimate fusion of antenna design. In such a fused configuration, the metasurface acts as the radiating aperture and can be combined with other EM functions, giving rise to many novel metantennas, such as reconfigurable intelligent metasurface antennas, topological optical phased array antennas, and integrated stealth communication antennas based on space–time coding [27,28]. Although metantennas feature compact and multifunctional characteristics, their design process is relatively complex due to the need for precise design of meta-atom arrangements and properties. For metasurface-assisted antennas, they have widespread applications and more flexible design approaches. For example, artificial magnetic conductor (AMC) structures can be flexibly utilized to replace ground planes, which not only maintains the simplicity of the overall antenna structure but also reduces the profile [12]. Additionally, partially reflective surface (PRS) structures can be employed as superstrates to enhance antenna directivity by creating a Fabry–Perot resonant cavity [29], while reflectarray and transmitarray metasurfaces offer beam-forming capabilities with low-profile configurations and high aperture efficiency [30]. Many dual-band Wi-Fi antennas were proposed with the assistance of an AMC [31,32,33,34,35]. For example, a novel dual-band AMC operating at 2.4 GHz and 5.8 GHz was proposed in [31]. After integration of a 4 × 4 AMC array, the peak gain of the original antenna increased from 1.65 dBi to 4.8 dBi at 2.4 GHz, and from 4.5 dBi to 7.75 dBi at 5.8 GHz, achieving a significant gain enhancement with a profile height of 9.2 mm. However, existing researchers still face challenges such as uneven gain distribution between the higher- and lower-frequency bands, along with integration concerns stemming from the antenna’s relatively large structural dimensions.

In light of the above challenges, a compact high-gain dual-band antenna with AMC reflectors is proposed in this paper. By replacing the ground plane with a 3 × 3 AMC array, miniaturized dimensions and high-gain radiation characteristics in operating bands of 2.42–2.48 GHz and 5.16–5.53 GHz are achieved. Compared to the original U-slot antenna, the size of the designed AMC-assisted antenna is reduced by 25%, while achieving high radiation gains of 7.65 dBi at 2.45 GHz and 8 dBi at 5.2 GHz. The proposed design effectively combines metasurface structures to reduce antenna dimensions and maximize radiation gain, indicating significant application potential in dual-band Wi-Fi, Bluetooth, MIMO, and other fields.

## 2. Methodology and AMC Structure Analysis

**Figure 1:**
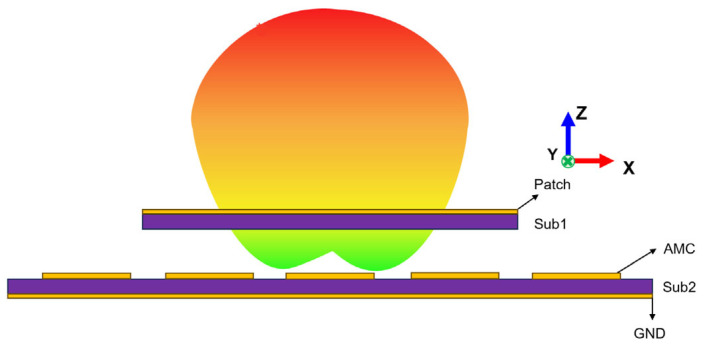
The schematic diagram of the AMC composite antenna.

An AMC is a type of metamaterial composed of periodic unit structures, with its main EM characteristic being a reflection phase of 0° and high surface impedance properties at resonant frequencies. When combined with antennas, an AMC offers multiple advantages, including low profile, excellent isolation, and high gain. The initial design of the AMC is a mushroom structure consisting of metallic patch grounded with a shorting via at the center of it [36]. It is known that a perfect electric conductor (PEC) has a reflection phase of 180° under normal incidence, while a perfect magnetic conductor (PMC), which does not exist in nature, has a reflection phase of 0° [37]. Another property of the AMC is high surface impedance, particularly in mushroom-type structures with connecting vias (PINs), which can create EM bandgaps to effectively suppress surface wave propagation. When used as an antenna reflector, this characteristic results in increased radiation gain and reduced back lobe level compared to common metal reflectors [38]. It should be noted that without these connecting vias, the dispersion diagram of an AMC structures does not exhibit any forbidden bandgap for surface wave propagation. Generally, a metal ground plane is used as a reflector to improve the radiation properties and increase the antenna gain. Such a metal ground plane behaves as a PEC with a 180° reflection phase, which leads to destructive interference between the reflected wave and the directly radiated wave when placed in close proximity to the radiation structure. This interference can be mitigated in conventional designs by increasing the separation distance between PEC ground and radiation pattern to approximately a quarter wavelength, but such an approach significantly increases the antenna profile [39]. In contrast, AMC structures are widely applied to replace the conventional metal ground plane as they provide in-phase reflection (0° reflection phase) while maintaining a low profile, thereby eliminating destructive interference and further improving the radiation properties of the antenna. Figure 1 shows the typical integration approach of an antenna with AMC structures. By applying the zero-phase reflection property of the AMC reflector, the structural dimensions of the antenna can be effectively reduced while enhancing the radiation gain.

**Figure 2:**
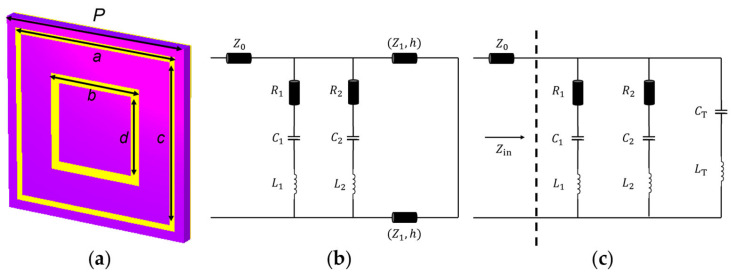
(**a**) The AMC unit, where *p* is the side length of the AMC unit cell, *a* and *c* are the outer edge length and inner edge length of the outer metal ring, *b* and *d* are the outer edge length and inner edge length of the inner metal ring; (**b**) equivalent circuit diagram of the AMC unit; (**c**) equivalent circuit with the transmission lines replaced by $L_{T}$ and $C_{T}$.

Based on the structural characteristics, the AMC can be classified into mushroom-type, planar-type, and multilayer type. Here, we use the planar type as an example to theoretically analyze the EM characteristics of AMC structures. Figure 2a shows a typical dual-band planar-type AMC based on the design proposed in reference [40], composed of a metal ground, dielectric substrate, and surface metal patterns. To achieve dual-band characteristics, the surface metal patches are designed as two concentric square metal rings, where the inner and outer rings resonate with the metal ground to operate at the higher- and lower-frequency bands, respectively. Figure 2b illustrates the equivalent circuit diagram of the planar AMC structure, which treats the AMC as a cascaded transmission line segment along the EM wave propagation direction. Each layer of metal and dielectric is considered as part of the transmission line. Specifically, the free space on one side of the AMC is modeled as a transmission line with characteristic impedance $Z_{0}=377\;\Omega$, the concentric double rings are equivalent to parallel RLC circuits, and the metal ground is a short line. In our previous research work, we have validated the effectiveness of the proposed equivalent circuit model for this planar double-ring AMC structure in the Sub-6G frequency band using the 2018 version of Advanced Design System (ADS) simulation software, with results showing that the phase response curves from circuit simulation and full-wave EM simulation almost completely overlap. The characteristic impedance of the dielectric substrate is $Z_{T}=\frac{Z_{0}}{\sqrt{\varepsilon_{r}}}$, where $\varepsilon_{r}$ is the relative dielectric constant of the substrate. As shown in Figure 2b, the dielectric substrate can be modeled by a transmission line of length *h*. The subwavelength transmission line segment can be represented by its equivalent circuit model, with series inductance $L_{T}=\mu_{0}\mu_{r}h$ and parallel capacitance $C_{T}=\frac{\varepsilon_{0}\varepsilon_{r}h}{2}$, where *h* is the spacing thickness, and $\mu_{r}$ and $\varepsilon_{r}$ are the permeability and dielectric constant of the dielectric spacer, respectively [41].

Therefore, the circuit in Figure 2b can be redrawn as Figure 2c. For the parallel equivalent circuit in Figure 2c, the input impedance can be expressed as

(1) $$Z_{in}=\frac{1}{\frac{1}{Z_{1}}+\frac{1}{Z_{2}}+\frac{1}{Z_{T}}},$$

(2) $$Z_{1}=R_{1}+j\omega L_{1}+\frac{1}{j\omega C_{1}},\;Z_{2}=R_{2}+j\omega L_{2}+\frac{1}{j\omega C_{2}},\;Z_{T}=j\omega L_{T}+\frac{1}{j\omega C_{T}}.$$

The input impedance equals zero, resulting in a higher-order equation in terms of *ω*. In practical applications, we can solve this equation using approximation methods [41]. The ω of different resonant frequencies can be expressed as

(3) $$\omega_{1}\approx \frac{1}{\sqrt{\left(L_{1}//L_{T})(C_{1}+C_{T}\right)}},$$

(4) $$\omega_{2}\approx \frac{1}{\sqrt{\left(L_{2}//L_{T})(C_{2}+C_{T}\right)}}.$$

On the other hand, fractional bandwidth (FBW) is also a critical parameter for evaluating the EM performances of the AMC elements. FBW not only quantifies the resonant frequencies of AMC structures but also directly determines their usable frequency range in practical applications. The theoretical foundation, calculation methodology, and physical significance of FBW are comprehensively elucidated in reference [41]. In dual-band AMC designs, the FBW of each operating band can theoretically be evaluated independently based on single-band AMC analysis methods. However, in practical implementations, EM coupling effects inevitably exist between the two frequency bands, resulting in shifts in their respective resonant frequencies, variations in bandwidth, and alterations in reflection phase characteristics. These coupling effects become particularly pronounced when the two operating bands are relatively close to each other. Consequently, during the design and optimization process of dual-band AMCs, it is imperative to comprehensively consider the mutual interactions between frequency bands, ensuring that each band meets the anticipated bandwidth requirements while maintaining structural compactness and practicality through precise EM simulations and parametric analyses.

From the above analysis, the optimization for AMC structures can be focused on three aspects, including frequency adjustment, bandwidth optimization, and coupling control. For frequency adjustment, the outer and inner rings correspond to low and high frequency responses, respectively. The operation frequency band can be effectively adjusted by changing the ring size, ring width, and gap of the rings, as well as the substrate thickness and dielectric constant. Bandwidth optimization can be achieved by increasing substrate thickness and appropriately increasing circuit losses (*R*_1_, *R*_2_), which can reduce the *Q* value, thereby broadening the bandwidth. With regard to the coupling, increasing the unit size and the distance between double rings can effectively reduce mutual coupling effects, thereby improving the overall performance of the AMC.

**Figure 3:**
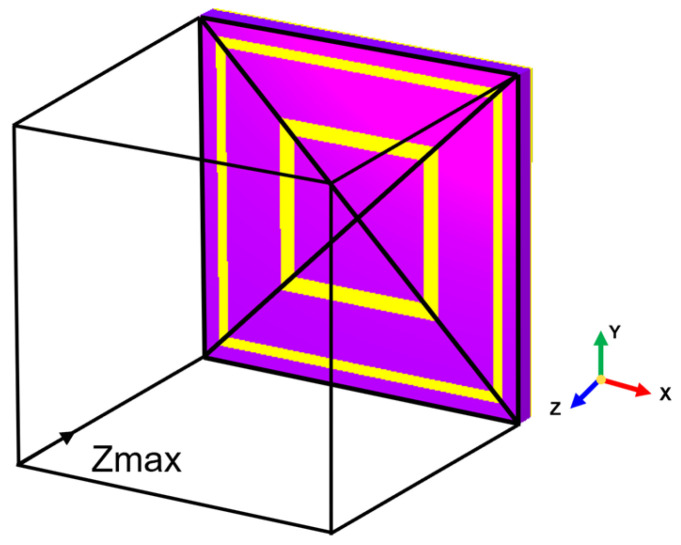
Simulation setup for the AMC unit.

**Figure 4:**
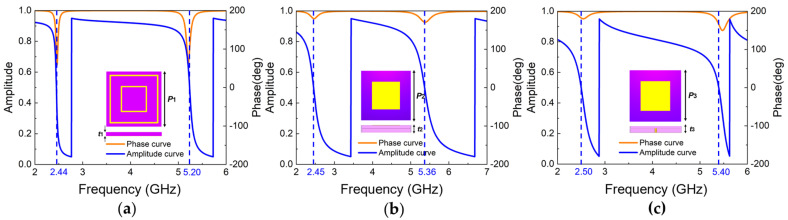
Simulation results and structural schematics of three AMC unit types: (**a**) planar-type; (**b**) double-layer patch structure; (**c**) mushroom-shaped.

Based on the theoretical analysis, simulations of various AMC types are conducted to investigate their EM performances in Wi-Fi frequency bands. Considering the attenuation during EM wave propagation, the reference plane should be placed exactly on the AMC surface to ensure the accuracy of the reflection phase, as shown in Figure 3. Figure 4 illustrates the geometric structures and simulation results of three types of AMC unit cells, including mushroom-shaped, planar, and multilayer structures. All AMC structures were designed using dielectric substrates with a relative permittivity (*ε_r_*) of 3.5 and loss tangent of 0.002. The unit cell periodicity (*p*) and substrate thickness (*t*) for each structure are specified in Table 1, along with their respective operating frequencies. In practical applications, the integration of AMC structures with antennas necessitates comprehensive consideration of multiple critical parameters. Among these, operating bandwidth (ΔF) and thickness (t) are two essential factors for evaluating AMC characteristics. The operating bandwidth determines the frequency range where the AMC can function effectively and align with the antenna’s operation frequency band, while the thickness directly impacts the profile height of the composite antenna. The fractional operating-bandwidth thickness (FOT), as a composite evaluation metric, elegantly integrates two key parameters into a dimensionless ratio, specifically, the ratio of relative bandwidth (ΔF/*f*_0_) to relative thickness (*t*/*λ*_0_). This formula enables FOT to simultaneously present both the bandwidth performance and physical dimension characteristics of AMC structures, thereby providing a more comprehensive and equitable platform for comparing different AMC configurations. A higher FOT value indicates that a wider relative bandwidth can be achieved per unit thickness, suggesting that the structure offers superior frequency coverage while maintaining a low profile. Consequently, when selecting and optimizing AMC structures for engineering applications, FOT serves as an integrated performance indicator that effectively guides the structural optimization as finding a balance between bandwidth requirements and spatial constraints. The relative bandwidth metric FOT is defined as follows:

(5) $$FOT=\frac{\Delta F/f_{0}}{t/\lambda_{0}}.$$

where ΔF is the operating bandwidth, and $\lambda_{0}$ is the wavelength corresponding to $f_{0}$.

Among the three types, planar AMC demonstrates significant advantages in terms of small size and low profile. Although the mushroom-shaped AMC exhibits superior FOT compared to the double-layer patch structure AMC, its structure is considerably more complex. The presence of copper pillars in the mushroom-shaped design introduces pronounced EM coupling phenomena, which in turn makes the antenna integration and fabrication more complicated. The planar-type structure, which has lower FOT, offers a better balance between EM performance and structural simplicity, especially the characteristic of low profile. Therefore, after comprehensive consideration of performance metrics, manufacturing complexity, and integration feasibility, the double-ring and double-layer patch structures are selected for the composite design of metasurface antenna.

## 3. Design of the Metasurface Antenna

**Figure 5:**
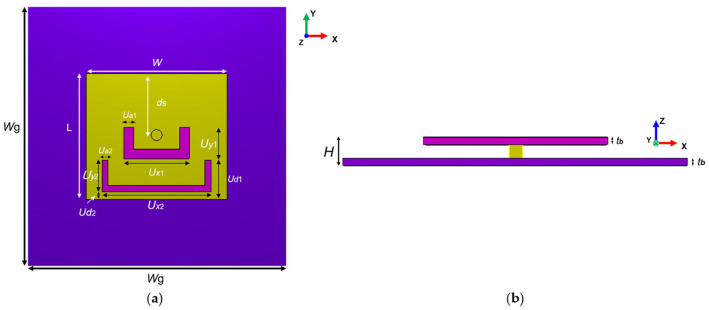
Original dual-band U-slot patch antenna: (**a**) top view of the antenna; (**b**) side view of the antenna.

Inspired by the design concept of the dual-band U-slot radiation pattern from reference [42], this paper presents a dual U-slot metasurface antenna for dual-band Wi-Fi applications. Compared to traditional multiband patch antennas, such as antennas with L-probe feeding, antennas with M-probe feeding, coaxial-fed stacked patch antennas, and aperture-coupled stacked patch antennas [43], this U-slot design method offers some significant advantages. Primarily, the proposed antenna employs a single-layer radiation patch structure, effectively eliminating the interlayer coupling effects commonly encountered in multilayer patch designs, while substantially reducing sensitivity to manufacturing tolerances. Additionally, the implementation of a straightforward coaxial feeding mechanism simplifies the feeding structure, which, in combination with the single-layer patch configuration, enables the antenna to achieve characteristics of compact size, low profile, and simplified construction. Figure 5 shows the basic structure of the antenna, which is divided into upper and lower layers with a total height of *H*. The upper layer consists of a dielectric substrate and patch with dimensions *W* × *L*, while the lower layer comprises a dielectric substrate and metal ground plane with dimensions *W*g × *W*g. The upper layer and bottom layer are spaced by an air layer with thickness of *h_a_*. The employed F4B substrate has a dielectric constant of 3 and a loss tangent of 0.002. Two U-shaped slots are cut into the patch to provide additional current paths.

**Figure 6:**
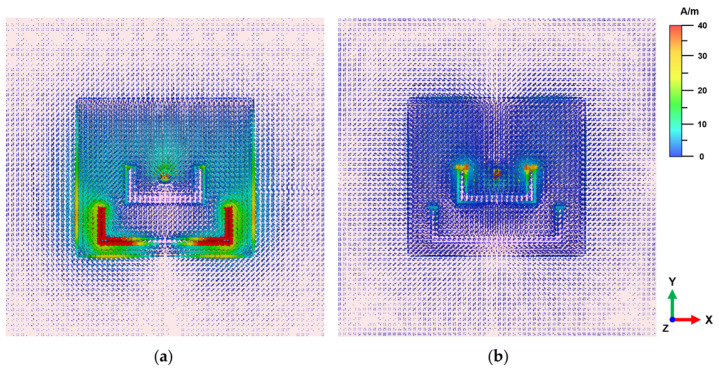
Surface current distributions on the antenna at different frequencies: (**a**) low frequency band (2.44 GHz); (**b**) high frequency band (5.2 GHz).

The design principle of the dual U-slot dual-band antenna is based on the mechanism of controlling surface current distribution through multiresonant structures. For a traditional rectangular patch without any slots, the surface current is mainly distributed along the length direction. The middle U-slots are introduced to primarily enrich the basic resonant modes of the antenna. The dimensions and position are optimized to introduce additional current path on the original patch, enabling the antenna to maintain good impedance matching over different frequency ranges. After feeding, the middle U-slots force the surface current to flow around the slot edges, forming extended current paths and impedance matching bandwidth. As shown in Figure 6a, the surface current mainly flows along the edges of the outer U-slot at the lower-frequency band, forming a longer current path. At the higher-frequency band, the current is mainly distributed along the inner slot, as shown in Figure 6b. The dominant current distributions introduced by U-slots correspond to the two operating frequency bands of the antenna, enabling the antenna to operate efficiently in two individual frequency bands while maintaining relatively stable radiation characteristics. It is worth noting that the surface current intensity at the lower-frequency band exhibits higher magnitude compared to that of the higher-frequency band. This differential current distribution characteristic is clearly visible in the surface current plots shown in Figure 6, further confirming the distinct operational modes of the dual U-slot antenna across its two operating frequency bands.

**Figure 7:**
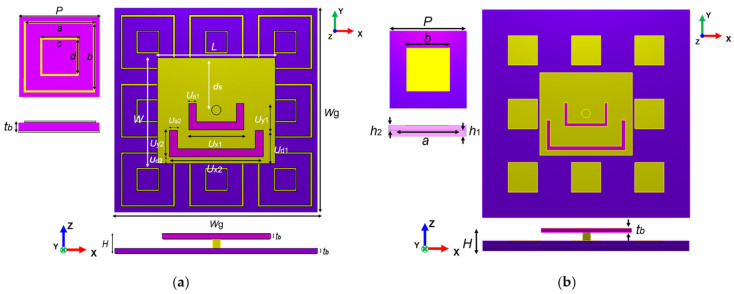
The structure of the composite antennas: (**a**) Antenna I, where *p* is the side length of the AMC unit cell, *a* and *c* are the outer edge length and inner edge length of the outer metal ring on the top of the AMC unit, *b* and *d* are the outer edge length and inner edge length of the inner metal ring; (**b**) Antenna II, where *p* is the side length of the AMC unit cell, *a* is the side length of the middle-layer metal patch of the AMC unit, and *b* is the side length of the top-layer metal patch.

**Figure 8:**
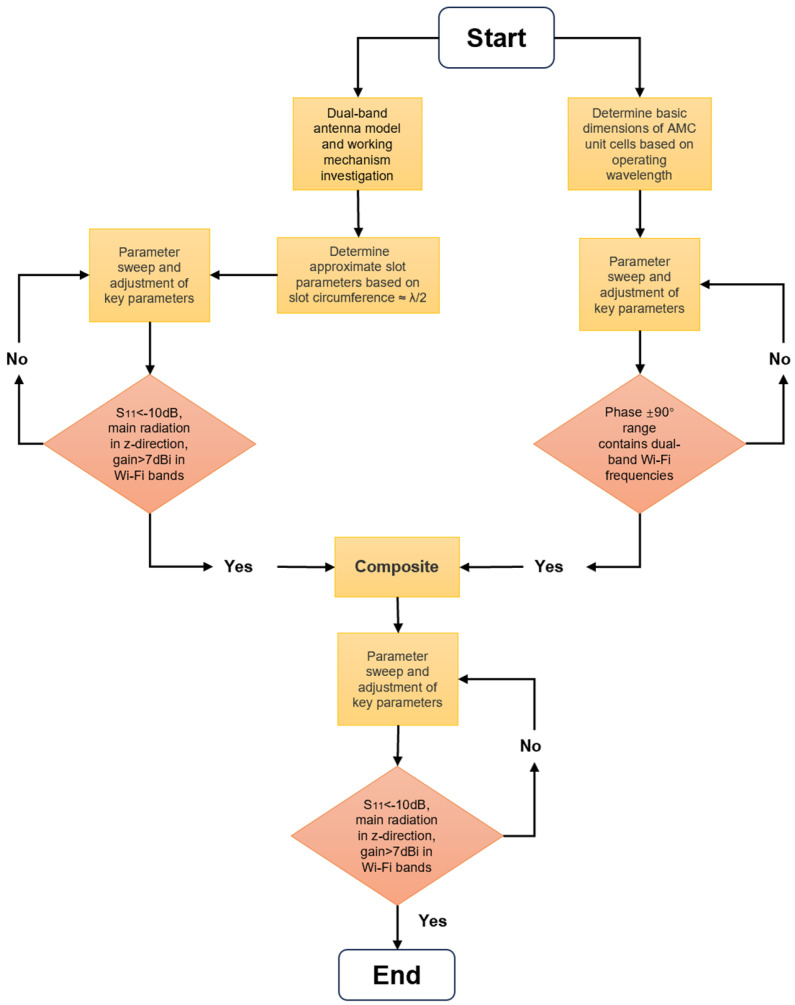
The flow chart of the antenna optimization process.

**Figure 9:**
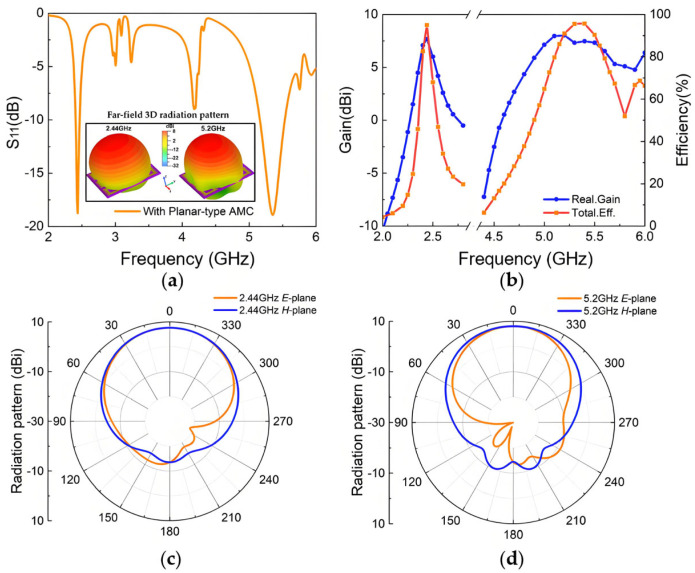
Simulation results of Antenna I: (**a**) reflection coefficient of the Antenna I and 3D radiation patterns at 2.44 GHz and 5.2 GHz; (**b**) realized gain and total efficiency; (**c**) radiation pattern at 2.44 GHz; (**d**) radiation pattern at 5.2 GHz.

**Figure 10:**
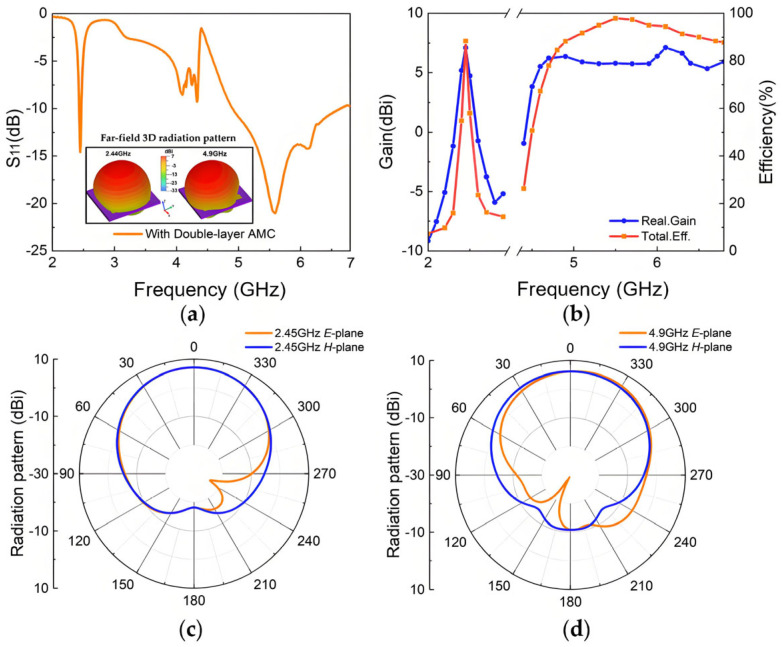
Simulation results of Antenna II: (**a**) reflection coefficient of the Antenna II and 3D radiation patterns at 2.45 GHz and 4.9 GHz; (**b**) realized gain and total efficiency; (**c**) radiation pattern at 2.44 GHz; (**d**) radiation pattern at 5.2 GHz.

To enhance antenna performance, the lower layer of the original antenna was replaced with an AMC array, as illustrated in Figure 7. The two AMC structures analyzed above are employed to design the metasurface antenna, including a single-layer dual-square-ring planar AMC structure and a double-layer square-patch AMC structure. Antenna I incorporates a 3 × 3 single-layer AMC array, while Antenna II incorporates an 8-unit double-layer AMC array with the center unit removed to avoid unwanted resonance peaks and improve impedance matching characteristics. The antenna optimization procedure is illustrated in Figure 8, where we employ parameter optimization methodology. The entire procedure consists of three critical parts: first, optimizing the original antenna structural parameters to achieve fundamental radiation characteristics; second, independently optimizing the AMC unit cell dimensions to realize the desired reflection phase properties; and, finally, integrating both components for comprehensive optimization of the composite dual-band antenna. This systematic optimization approach can be applied for targeted enhancement of performance metrics (such as bandwidth expansion in the 2.4 GHz band) according to application requirements. After multiple parameter optimization iterations and adjustments, the parameters of the designed metasurface antennas are determined, with detailed values presented in Table 2. Figure 9 and Figure 10 illustrate the reflection coefficients, gain efficiency, and both 3D and 2D radiation patterns of the metasurface antennas. As depicted in Figure 9a, Antenna I achieves impedance bandwidths of 2.42–2.48 GHz (2.5%) and 5.16–5.53 GHz (6.9%) at reflection coefficient |S11| < −10 dB, with the main radiation lobe directed along the positive z-axis. Figure 9b reveals that the antenna reaches high gains of 7.65 dBi and 8 dBi in its two operating bands, with total efficiency above 90%. The radiation patterns displayed in Figure 9c,d demonstrate perfect matching in the forward radiation along the positive z-axis. Similarly, Figure 10 demonstrates that Antenna II operates at 2.43–2.48 GHz (2.1%) and 4.9–6.64 GHz (30.2%), achieving a significantly wider bandwidth in the higher-frequency band. The dual-band gains are 7.1 dBi and 6.44 dBi, respectively, with efficiency around 88%. At the higher-frequency band (around 5.5 GHz), Antenna II exhibits an exceptional total efficiency of 97.8%. The phenomenon of higher total efficiency yet lower gain compared to Antenna I can be attributed to a slight deviation of approximately 5° in the main lobe direction and additional energy dissipation in the side lobes. This trade-off between efficiency and directional gain demonstrates the complex performance characteristics of different metasurface configurations.

Table 3 shows the performance comparison of the two composite antennas. The results indicate that the planar AMC effectively reduces the antenna size and achieves high gains of 7.65 dBi and 8 dBi at 2.44 GHz and 5.2 GHz, respectively. However, the double-layer patch AMC has a broad bandwidth of 4.9–6.64 GHz at 5.2 GHz with a relatively larger size. From the simulation results, the two types of AMC structures have different comparative advantages. The double-layer patch AMC achieves a wider frequency band while sacrificing profile height, whereas the planar AMC simultaneously achieves high gain and low profile but with relatively narrow bandwidth. This actually reflects the trade-off between bandwidth and other metrics (such as profile height, gain, and size). As our primary focus is on high-gain and miniaturization characteristics, Antenna I is more suitable for integrated designs in Wi-Fi modules; therefore, we subsequently investigated its radiation performance in experiments.

## 4. Results and Discussion

**Figure 11:**
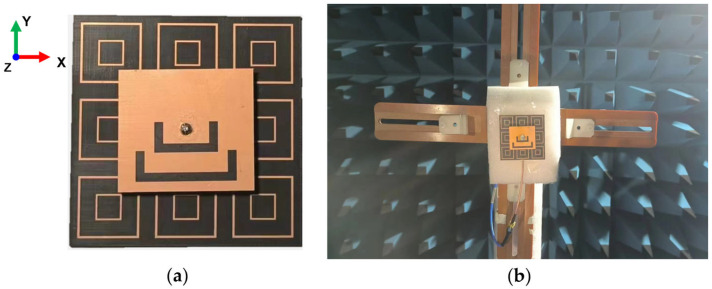
The fabricated Antenna I and measurement scenario: (**a**) photograph of the Antenna I; (**b**) measurement scenario in the semi-anechoic chamber.

The sample of Antenna I is precisely fabricated by commercial print circuit board (PCB) technology, with overall error controlled within approximately ±0.02 mm. The dielectric layers employ F4B substrates, while the metal layers are printed copper foil with 0.035 mm thickness. To ensure the stability of the feeding, the via hole at the upper layer is covered with copper, and the location of the coaxial feed point is optimized to achieve optimal impedance matching. Experiments are conducted in a semi-anechoic chamber, as shown in Figure 11, applying a Ceyear 3656B vector network analyzer to measure S-parameters, and a far-field test system to measure radiation patterns.

**Figure 12:**
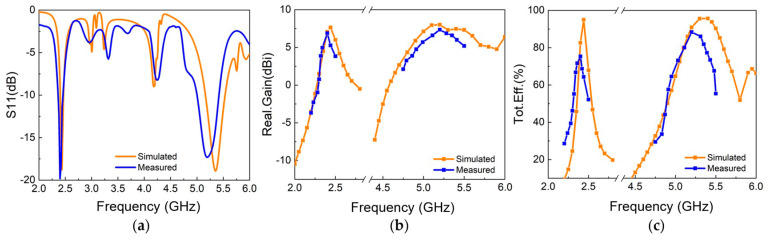
Measurement and simulation results of Antenna I: (**a**) reflection coefficient; (**b**) realized gain; (**c**) total efficiency.

**Figure 13:**
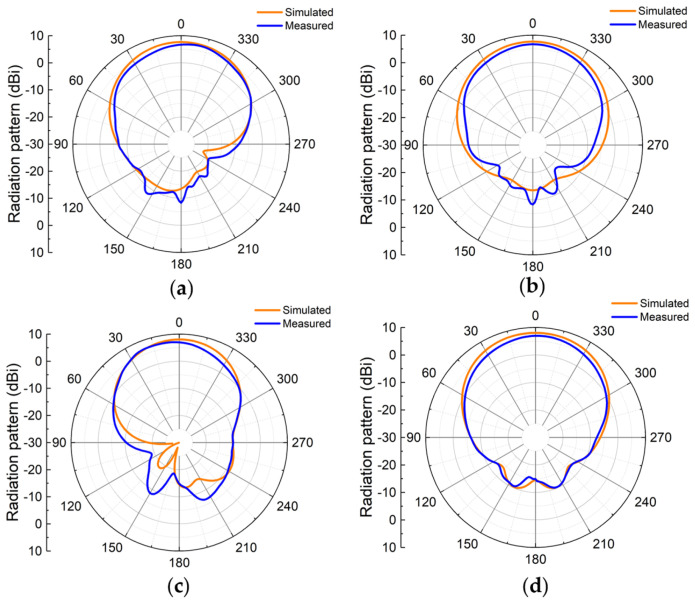
Radiation patterns of Antenna I in experiment and simulation: (**a**) 2.44 GHz *E*-plane; (**b**) 2.44 GHz *H*-plane; (**c**) 5.2 GHz *E*-plane; (**d**) 5.2 GHz *H*-plane.

As shown in Figure 12a, the measurement results indicate that the antenna achieves good impedance matching (|S11| < −10 dB) in two frequency bands: 2.37–2.45 GHz (3.3%) and 5–5.4 GHz (7.8%). Figure 12b demonstrates that Antenna I exhibits stable radiation characteristics in both operating bands, with maximum gains of 6.67 dBi and 7.14 dBi at the lower-frequency band (2.44 GHz) and higher-frequency band (5.2 GHz), respectively, with deviations from simulation results less than 1 dBi. Figure 12c shows that the measured efficiency in both frequency bands has decreased, possibly due to the fabrication losses in the actual prototype. With regard to the radiation patterns displayed in Figure 13, the measured results show high consistency with simulations, while the main lobe in the lower-frequency band exhibits a small deviation of about 3°. The radiation patterns presented in Figure 13 display absolute values (not normalized) for both simulated and measured results. For the lower-frequency band (2.44 GHz), the simulated side lobe level is −13.5 dBi while the measured value is −8.35 dBi, with front-to-back ratio of 21.26 dB in simulation and 15.03 dB in measurement. For the higher-frequency band (5.2 GHz), the simulated side lobe level is −14.64 dBi and the measured value is −14.78 dBi, with a front-to-back ratio of 22.64 dB in simulation and 21.78 dB in measurement. The differences between measured and simulated results are primarily attributed to manufacturing tolerances, the influences of feeding connectors, and the measurement environment.

Table 4 presents a comprehensive comparison between the proposed antennas and previously reported dual-band AMC antennas. Antenna I demonstrates exceptional performance with remarkably consistent high gains of 7.65 dBi and 8 dBi at 2.44 GHz and 5.2 GHz, respectively. This uniform gain distribution across both operating bands represents a significant advantage over most reference designs that exhibit substantial gain variations between two frequency bands. Furthermore, Antenna I achieves outstanding total efficiencies of 95% and 95.7% at the respective operating frequencies. Antenna II achieves a relative bandwidth of 30.2% in the 4.9 GHz frequency band, which is approximately two times wider than that of conventional dual-band AMC antennas. This exceptional bandwidth is complemented by commendable gains of 7.1 dBi and 6.44 dBi at 2.45 GHz and 4.9 GHz, respectively, along with excellent total efficiency values of 88.4% and 88.3%.

The proposed AMC-based antenna maintains competitive factors while delivering superior EM performances. These characteristics make it highly suitable for modern wireless communication systems that require dual-band EM responses, including Wi-Fi 6 applications (2.4/5 GHz), Bluetooth connectivity, and MIMO systems for enhanced data throughput. The exceptional radiation efficiency also suggests potential benefits for energy-efficient wireless devices and extended battery life in portable applications.

## 5. Conclusions

This paper presents a high-gain dual-band metasurface antenna based on AMC structures, effectively resolving the design contradiction between gain balance and structural compactness in traditional dual-band antennas. Theoretical analysis of the AMC structures was conducted to obtain the guidelines of dual-band characteristic and broad bandwidth. Two types of AMC structures were selected to design the metasurface antenna, including dual-ring planar type and double-layer patch type. As a result, the dual-ring planar AMC structure significantly enhanced the radiation gain while maintaining low-profile characteristics, achieving stable gains of 7.65 dBi and 8 dBi in the 2.4 GHz and 5 GHz bands, respectively. The double-layer patch AMC structure successfully expanded the bandwidth of the higher-frequency band to 30.2% by enhancing coupling effect between cascading structures. Experimental results indicate that the designed antenna exhibits good radiation performances in terms of impedance bandwidth, dual-band radiation efficiency (>90%), broad radiation angle, and radiation gains across the operation frequency bands. Compared with existing dual-band AMC antennas, this design demonstrates significant advantages in high gain, miniaturization, and high efficiency. The proposed antenna provides a reliable solution for highly integrated wireless communication modules that has great potential for the applications in Wi-Fi 6, Bluetooth, and MIMO technology.

## Figures and Tables

**Table 1:** Performance comparison of three AMC unit types.

AMC	Operating Bandwidth/GHz	FOT	Unit Cell Dimension (*P* × *P*)/mm^2^	Substrate Thickness (*t*)/mm
Planar-type	2.42–2.48	2	21 × 21	1.5
	5.15–5.25	0.74		
Double-layer patch structure	2.32–2.57	3.59	24 × 24	3.5
	5.17–5.53	1.08		
Mushroom-shaped	2.30–2.60	5	24 × 24	3
	5.00–5.50	1.81		

**Table 2:** Antenna parameters.

Antenna	*W*g	*W*	*L*	*H*	*d_s_*	*t_b_*	*R*	*U_a_*_1_	*U_d_*_1_	*U_x_*_1_	*U_y_*_1_
I	33	37.9	33.8	5.5	16.7	1.5	1.5	2.5	10.8	17.7	8.5
II	36	32	28.6	8	14.1	1.5	1.5	1.1	10	14.9	8.1
**Antenna**	***U_a_*** **_2_**	***U_d_*** **_2_**	***U_x_*** **_2_**	***U_y_*** **_2_**	**p**	**a**	**b**	**c**	**d**	***h*** **_1_**	***h*** **_2_**
I	2.7	2.1	30.2	8.5	22	17.5	16.2	7.4	6.4	--	--
II	1.4	1.7	27.4	10.5	22	16.1	10.2	--	--	2	1.5

**Table 3:** Performance comparison of the metasurface antennas.

Antenna	Operation Bandwidth/GHz	Observation Frequency /GHz	Gain/dBi	Dimension/mm^3^
I	2.42–2.48	2.44	7.65	66 × 66 × 5.5
	5.16–5.53	5.2 GHz	8	
II	2.43–2.48	2.45 GHz	7.1	72 × 72 × 8
	4.9–6.64	4.9 GHz	6.44	

**Table 4:** Performance comparison with some existing dual-band AMC antennas.

Ref.	Frequency (GHz)	Bandwidth (%)	Realized Gain (dBi)	Total Efficiency (%)	Size (mm^2^)	AMC Type	Substrate Type
[31]	2.4/5.8	13/10.2	4.8/7.75	N.A.	49 × 49	Planar AMC	Rogers RO 3003
[32]	2.4/5.8	11/15.5	8.06/7.35	N.A.	57 × 57	Planar AMC	FR-4
[33]	2.45/5.8	9.6/12.4	4.88/4.73	N.A.	44.4 × 44.4	Planar AMC	FR-4
[34]	2.4/4.7	5.3/9.6	5/7.5	N.A.	79.9 × 79.9	Planar AMC	FR-4
[35]	2.45/5.8	1.55/3.5	2.44/6.17	50/72	28.81 × 19.22	Planar AMC	FR-4
[40]	2.45/5.8	20.8/16.5	7.02/4.23	91.3/85.8	102 × 102	Planar AMC	Felt
Antenna I	2.44/5.2	2.5/6.9	7.65/8	95/95.7	66 × 66	Planar AMC	F4B
Antenna II *	2.45/4.9	2.1/30.2	7.1/6.44	88.4/88.3	72 × 72	Double-layer AMC	F4B

* Simulation.

## Data Availability

The original contributions presented in the study are included in the article; further inquiries can be directed to the corresponding author.
